# Bilateral Paget's Disease of the Breast—Case Report of Long-Time Misdiagnosed Tumors with Underlying Ductal Carcinomas and Review of the Literature

**DOI:** 10.1155/2014/152836

**Published:** 2014-03-03

**Authors:** Dietrich Barth

**Affiliations:** Hautarztpraxis Leipzig/Borna, Rudolf Virchow Straße, Borna, 04552 Leipzig, Germany

## Abstract

Paget's disease of the breast is often misdiagnosed. We report on a 72-year old patient with a history of 2.5 years without any malignant findings, followed by the identification of a bilateral Paget's disease with bilateral breast cancers. This case underlines how important histological examinations even in unusual clinical pictures are.

## 1. Introduction

Paget's disease (PD) of the breast can be a diagnostic challenge. It might take years until the diagnosis. If the skin changes are intended to be benign but do not respond to topical therapy, a biopsy has to be performed to exclude malignancies. Almost all cases are single sided. We observed one of the rare cases of bilateral PD.

## 2. A Case Report

**Figure 1:**
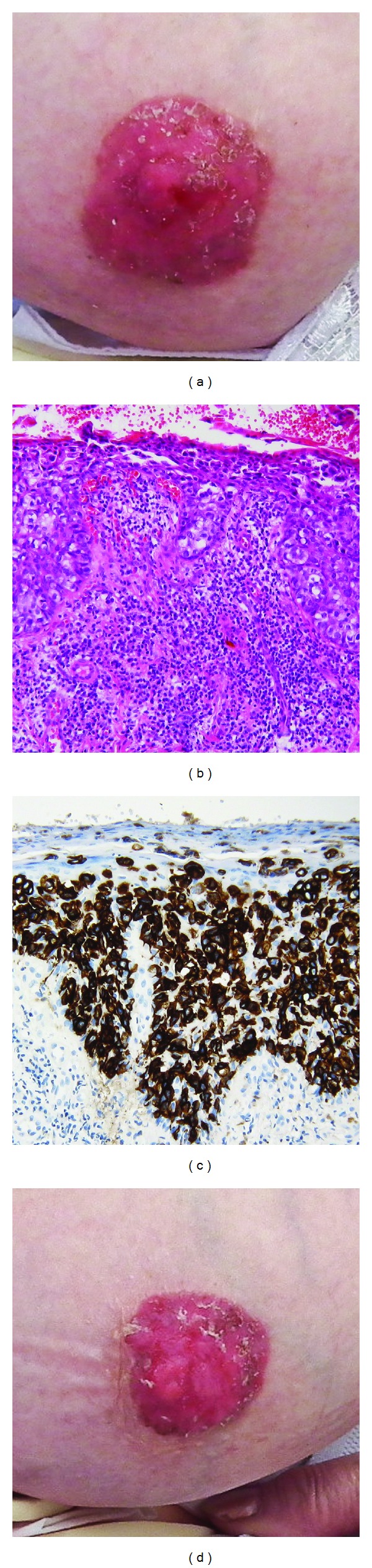
Clinical picture at time of first presentation and histological stains ((a) right breast, (b) cytokeratin 7 stain, (c) HE stain, and (d) left breast).

A 72-year-old woman (para 1) was seen with erythematous and eczematous patches that developed simultaneously on both nipples and had been present for 2.5 years (Figures 1(a) and 1(d)). No individual or familiar risk factors were known. She was extensively evaluated by gynecology and several investigations were performed (mammography, vacuum-punch biopsies, and cytological examination of breast fluid), but only minor dysplastic changes were detected in the breast fluid cytology. The patient was then treated with topical antimycotics, antibiotics, and corticosteroids.

After 2.5 years she was referred to dermatology, where we biopsied both nipples. The histopathology showed epidermal cells with hyperchromatic and polymorphic nuclei, intraepithelial gland cells (Figure 1(b)), and a high expression of cytokeratin 7 (Figure 1(c)), so-called Paget's cells. Cytokeratin 7 is a typical marker for glandular and transitional epithelia.

Because of an induration of the left mamma and the incidence of underlying carcinomas, the patient was evaluated again by gynecologists who decided to operate on both breasts. They identified a bifocal invasive ductal carcinoma and an intermediate grade ductal carcinoma in situ (DCIS) of the left breast and a low-grade DCIS of the right central breast. Sentinel lymph nodes were not involved. Following surgery, the patient received chemotherapy with 6 cycles FEC (5-fluorouracil, epirubicin, and cyclophosphamide), trastuzumab, because of positive Her-2 status, radiotherapy, and tamoxifen. At 1.5-year follow-up being maintained on tamoxifen, she showed no relapse.

## 3. Discussion

Between 1 and 4% of all breast cancers are Paget's diseases [1]. Bilateral synchronous tumors occur in about 1% of all breast cancers [2]. So far there are less than 10 reported women with synchronous bilateral PD. The age of these patients ranges from 45 to 74 years [3, 4]. This phenomenon has been described twice in men [5, 6].

The disease appears in three forms: (1) associated with an underlying ductal carcinoma in situ (DCIS), (2) associated with an invasive carcinoma, or (3) without any underlying malignancy [7]. The majority of patients with PD have an underlying DCIS or even invasive carcinoma [8, 9].

For bilateral PD the limited data are controversial. Sahoo et al. [4] connected the PD of its patient to an underlying lobular CIS because of the immunohistochemical profile; Xie et al. [10] found no underlying tumor, whereas the patients of Anderson [11] and Franceschini et al. [12] as well as our patient had underlying ductal carcinomas.

It is still in discussion if the disease is the cause or consequence of an underlying malignancy. Most authors support the epidermotropism of malignant ductal gland cells into the epidermis. Some favor the migration of malignant keratinocytes from epidermis into deeper tissues, because in up to 50% of the cases no underlying tumors can be found [13].

Multiparous patients seem to have a reduced risk of ductal, lobular, tubular, and mucinous breast cancers. By contrast, the risk of medullary breast cancers increases with the number of pregnancies [14]. If similar findings for PD are evident [15] should be subject for further investigations. One explanation could be the inflammatory processes and restructuring of the ductal network after lactation [16].

The cause for the bilateral form of PD remains uncertain as the number of patients is limited and the reported patients differ in age, gender, and ethnicity (see Table 1). Coincidences cannot be excluded.

The treatment options are mastectomy or breast-conserving strategies including nipple excision or central lumpectomy with a lymph node biopsy. There are reports of positive lymph nodes even without any underlying malignancies [8, 9]. If surgery is not possible, radiotherapy, laser therapy, photodynamic therapy, or chemotherapy, for example, with trastuzumab or imiquimod, can offer a therapeutic alternative.

Although radiological diagnostic tools have improved over the years, each suspicious skin lesion of the breast must be biopsied in order to avoid the progression of a malignancy. Our patient's history of 2.5 years without any findings underlines the importance of early histological examinations.

## Figures and Tables

**Table 1:** Summary of all available cases of bilateral Paget's disease.

Age/gender	Associated cancer	Country	Author/reference
53/female	L: intraductal carcinoma	USA	Anderson 1979/[11]
Female	?	Netherlands	Knol and Voorhuis 1981/[17]
Female	?	India	Sinha and Prasad 1983/[18]
Male	?	India	Nagar 1983/[5]
74/female	?	Portugal	Fernandes et al. 1990/[3]
Female	?	Greece	Markopoulos et al. 1997/[19]
53/female	R: LCIS of the nipple, DCIS + microinvasive ductal carcinoma L: LCIS of the nipple, DCIS	USA	Sahoo et al. 2002/[4]
73/female	R: high-grade intraductal carcinoma L: micropapillary invasive carcinoma	Italy	Franceschini et al. 2005/[12]
74/male	R: infiltrative ductal carcinoma	Turkey	Ucar et al. 2008/[6]
45/female	None	China	Xie et al. 2012/[10]
72/female	R: low-grade DCIS L—intermediate DCIS, invasive ductal carcinoma	Germany	Barth 2014

L: left breast; R: right breast; DCIS: ductal carcinoma in situ; LCIS: lobular carcinoma in situ.
